# Characterization of Typical Behaviors of Mares in the Opening Phase of Parturition—Influence of Parity and Dystocia

**DOI:** 10.3390/ani14071036

**Published:** 2024-03-28

**Authors:** Hannah Lindinger, Axel Wehrend

**Affiliations:** Veterinary Clinic for Reproductive Medicine and Neonatology, Justus Liebig University, 35392 Giessen, Germany

**Keywords:** mare, parturition, behavior, birth monitoring

## Abstract

**Simple Summary:**

Birth monitoring in the mare is crucial for preventing potential losses. To enhance the efficacy of birth monitoring and facilitate the development of monitoring programs rooted in behavioral parameters, we analyzed mare behavior during stage I of parturition. Our investigation revealed distinct behavioral patterns among mares, highlighting considerable variability among individuals. Interestingly, we observed correlations between parity, the birth process, and behavior during stage I of parturition, suggesting significant influences on the mare’s behavior during this critical period.

**Abstract:**

The identification of typical behaviors in stage I of parturition, the opening phase, can be used to improve birth monitoring in the mare. Therefore, this study aimed to comprehensively analyze mare behavior during the opening phase. Real-time recordings of 66 births involving 56 warmblood mares were analyzed using camera footage. Behaviors such as increased locomotor activity, pawing with front hooves, lifting the tail, rolling, kicking with the hind legs toward the abdomen, and looking at the abdomen increased significantly (*p* < 0.001) in the four hours preceding parturition. Within the last hour of the observation period, a statistically significant change was observed for the duration of lying in the sternal and lateral position (*p* < 0.001). Significant correlations were observed between parity and the total number of repetitions of lying in the sternal position (*p* < 0.05). Furthermore, the birth process influenced the repetitions of lying in the lateral position (*p* < 0.05). These findings indicate distinct behavioral patterns during the opening phase of parturition, which were evident across the observed mares. Nonetheless, notable individual differences were also identified among the mares.

## 1. Introduction

The gestation length in mares varies from 320 to 360 days [1]. Predicting the exact time of parturition is challenging due to the subtle nature of physical changes such as sinking of the pelvic ligaments, udder formation, or vulva edematization. These changes are barely or only slightly visible compared to other domestic mammals and are subject to strong individual differences in terms of appearance and temporal changes [2].

The significance of birth monitoring and the accurate prediction of parturition lies in the timely intervention necessary for dystocia, thus averting potential illness or mortality of the mare and foal and ensuring timely colostrum intake by the foal [3,4,5,6].

Dystocia, accounting for two to thirteen percent of mare deliveries [7,8], is considered an emergency, and rapid intervention is crucial for the survival of the foal. 

The behavior of the mares changes shortly before parturition. Shaw et al. [9] noted minimal behavioral shifts in mares during the days leading up to parturition compared to earlier stages of gestation, with significant changes occurring only on the night of parturition. Increased locomotor activity and recurrent lying down followed by immediate standing up are characteristic behaviors observed during the opening phase of parturition [2,9,10,11,12,13,14,15]. Other behaviors observed in proximity to the time of parturition are weight shifting from one front leg to the other [2,11], reduced feed intake [2,9,15], increased lying in the lateral position [11,15], pressing the hindquarters against the stall wall, rubbing the perineal area [10,11], looking at the abdomen [10,11,15], and frequent urination in small amounts [13,15]. Jeffcott [2] also describes localized sweating and frequent defecation in small amounts. Auclair-Ronzaud et al. [10] noted increased tail activity and flehmen in mares before parturition, while Jung et al. [15] observed mares exhibiting pawing with their front hooves and lowering their heads without eating. 

Previous studies on mare behavior in proximity to the time of parturition have typically focused on individual or a limited number of predetermined behaviors, resulting in an incomplete understanding of mare behavior during stage I of parturition. Therefore, the aim of this study is to analyze the behavior of mares in stage I of parturition in more detail and to assess whether the number of animals performing certain behaviors and the frequency with which certain behaviors are performed changes as parturition approaches. 

A detailed examination of the behavior in the final hours before foal expulsion has the potential to enhance existing birth monitoring systems, particularly if artificial intelligence is employed for birth detection.

## 2. Materials and Methods

### 2.1. Animal Cohort

A total of 86 parturitions involving 70 warmblood mares were monitored during the 2021 and 2022 foaling seasons, of which 12 mares were maiden mares and 54 mares were pluriparous. The average age of the mares was 11.5 ± 4.7 years (mean ± SD), ranging from four to 22 years. Their gestation period averaged 342 ± 9.3 days (mean ± SD), varying from 325 to 365 days. Complete video recordings of the last four hours of 66 out of the 86 parturitions were recorded. Video recordings of eight mares were obtained during both years. 

The study was conducted at a veterinary practice for horses located in Oldenburg Münsterland, Lower Saxony, Germany.

### 2.2. Ethical Basis

Mare birth monitoring was conducted at the owners’ request as part of the veterinary service. The mares were cared for by the staff of the veterinary practice in accordance with the animal husbandry guidelines of the Federation of Animal Society [16]. The evaluation of the parameters collected during birth monitoring does not require ethical approval under the German Animal Welfare Act. 

### 2.3. Camera Surveillance

Foaling occurred in camera-monitored 6 × 4 m and 4 × 3 m boxes under the supervision of a veterinarian. Three cameras were installed in the foaling stall, allowing the stall boxes to be viewed in their entirety. Cameras from ieGeek (Hong Kong, model 1080P), which have a motion sensor, were used. Continuous recordings were made of the mares within 30-min time frames during the entire time. There was no pause between the recordings, which meant that the mares’ behavior could be evaluated without interruption. The cameras were connected to a Synology Network Attached Storage (NAS) via Wi-Fi, with surveillance managed and recorded using Synology’s “Surveillance Station” software 9.0, and accessed through the “DS CAM” app from Synology Inc. (Taipeh, Taiwan). 

### 2.4. Criteria for Video Evaluation

Prior to the commencement of the study, a set of criteria was established to assess the video footage. This was used to retrospectively analyze and evaluate the behavior of the mares during stage I of parturition. The end point of the observation period was reached when the amniotic sac or fetal parts between the rima vulvae were visible. This point in time was chosen because the rupture of the amniotic sac or the associated discharge of the amniotic fluid (beginning of stage II of parturition) could not be reliably detected on the video recordings. The observation period was determined by going back four hours from this end point.

At first, mare behavior was categorized into three superordinate behaviors: lying, standing, and walking. 

These three superordinate behaviors were further subdivided and the following behaviors were differentiated:

Walking mare: locomotor activity (defined as the visible intentional forward movement regardless of speed or duration); 

Recumbent mare: Lying in sternal position, lateral position, or rolling;

Standing mare: Engaging in food/water intake, kicking with hind legs towards the abdomen, pawing with front hooves, exhibiting flehmen/yawning/chewing, looking towards the abdomen, lifting the tail, tail slapping, rubbing the rear body region/perineal area against the stall wall, defecation/urination, or exhibiting resting behavior.

**Figure 1:**
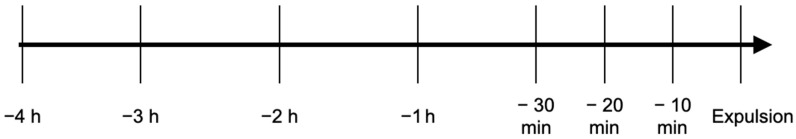
Evaluation intervals of mare video recordings in the last four hours before foal expulsion. The beginning of expulsion was determined by the initial visibility of the amniotic sac between the mare’s labia. Intervals were delineated as follows: from −4 h to −1 h, each comprising one hour. The final hour of observation was segmented into one 30-min interval and three 10-min intervals. The division of the last hour into smaller intervals was made because changes in behavior during this period are considered to be of greater importance for the detection of parturition.

The behavior was analyzed over four hours before the start of the expulsion. This period was further differentiated in order to be able to depict changes in behavior closer to the time of expulsion (Figure 1).

The four intervals of the last hour of the observation period (one 30-min and three 10-min intervals) were summarized as the last hour of the observation period (1 h—expulsion) to provide an overview of whether a change occurs in proximity to the time of parturition. For a more precise evaluation of this period, it was subdivided into the intervals mentioned above, shown in Figure 1.

For behaviors exhibiting prolonged durations, the total time spent performing each behavior was recorded within the specified time intervals. This method applied to behaviors such as locomotor activity, lying in the sternal position, lying in the lateral position, food/water intake, and resting behavior. Conversely, for all other behaviors, only the repetitions of occurrence were documented.

To analyze the potential influence of the birth process, a division was made into two groups: eutocia (n = 56) and dystocia (n = 10). Reasons for the presence of dystocia are as follows: premature placental abruption intra partum (n = 5), incorrect positioning (n = 3), incorrect posture (n = 1), and constriction of the caudal soft birth canal (n = 1). Additionally, the influence of parity was examined by differentiating between maiden mares (n = 12) and pluriparous mares (n = 54).

### 2.5. Statistical Analysis

The data were collected using Microsoft Excel Office software (version 16.70, Microsoft Cooperation, Redmond, WA, USA). Prism 9.5.1 software (GraphPad, Boston, MA, USA) and Microsoft Excel Office 10 were employed for results analysis, and graphical representations were generated using Prism 9 software. 

To explore whether behaviors undergo changes in expression as parturition approaches, a paired t-test was conducted for datasets exhibiting a normal distribution. For non-normally distributed datasets, a one-way analysis of variance (ANOVA) with the Kruskal–Wallis test served as a post-hoc analysis to examine behavioral changes over time. The influence of parity and birth process on behavior within specific time intervals was assessed using t-tests with Mann–Whitney tests as post-hoc analyses. For multiple comparisons concerning the influence of parity over time on behavior, a two-way ANOVA with Šidák test was employed as a post-hoc test. Differences were considered significant if *p* < 0.05.

## 3. Results

### 3.1. Lying Behavior

**Figure 2:**
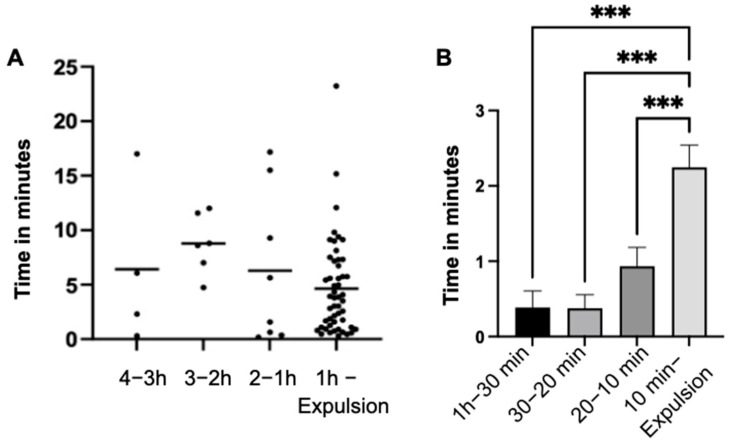
Duration and average lying time in minutes in the sternal position of the mares (n = 66) across observation time intervals (**A**). Lying time with the standard error of the mean value of the mares (n = 66) in sternal position during the intervals of the last hour of the observation period (**B**). (significance level: ***: *p* < 0.001).

The mean values of the duration lying in the sternal position did not exhibit significant changes between the individual observation intervals (*p* = 0.143) throughout the entire observation period (Figure 2A). However, a notable increase in lying time was observed (*p* < 0.001) during the last ten minutes of the observation period compared to the three previous intervals (Figure 2B). Additionally, the number of mares lying down significantly increased (*p* < 0.001) from 22 (20–10 min) to 48 (10 min—expulsion).

**Figure 3:**
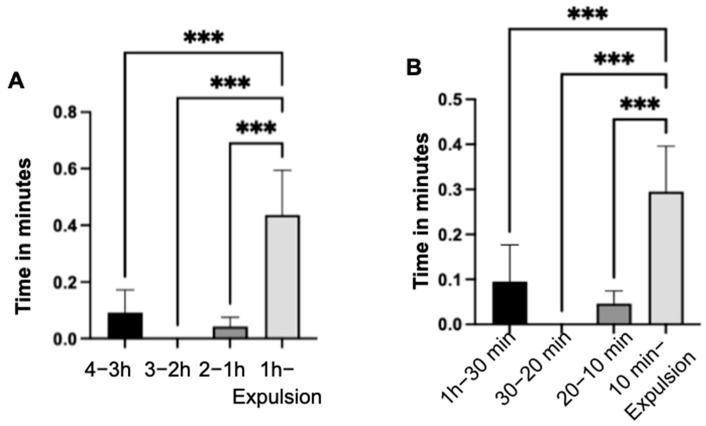
Lying time in minutes of the mares (n = 66) in lateral position with indication of the standard error of the mean value in the indicated time intervals (**A**) and in the intervals of the last hour (**B**) of the observation period. (significance level: ***: *p* < 0.001).

In the last hour of the observation period, a highly significant (*p* < 0.001) increase in lying time in the lateral position was detected compared to the three previous intervals (4–3 h; 3–2 h; 2–1 h) (Figure 3A).

Specifically, when analyzing the last hour independently, a highly significant (*p* < 0.001) increase in lying time in the lateral position was noted in the three preceding intervals (1 h–30 min; 30–20 min; 20–10 min) compared to the last ten minutes (10 min–expulsion) of the observation period (Figure 3B). 

During the entire observation period, 33.3% of the mares were observed lying on their sides. In the last ten minutes of the observation period, 20 mares showed the behavior. This reflects a highly significant increase (*p* < 0.001) in the number of mares in this specific time interval compared to the preceding one (20–10 min).

### 3.2. Locomotor Activity

**Figure 4:**
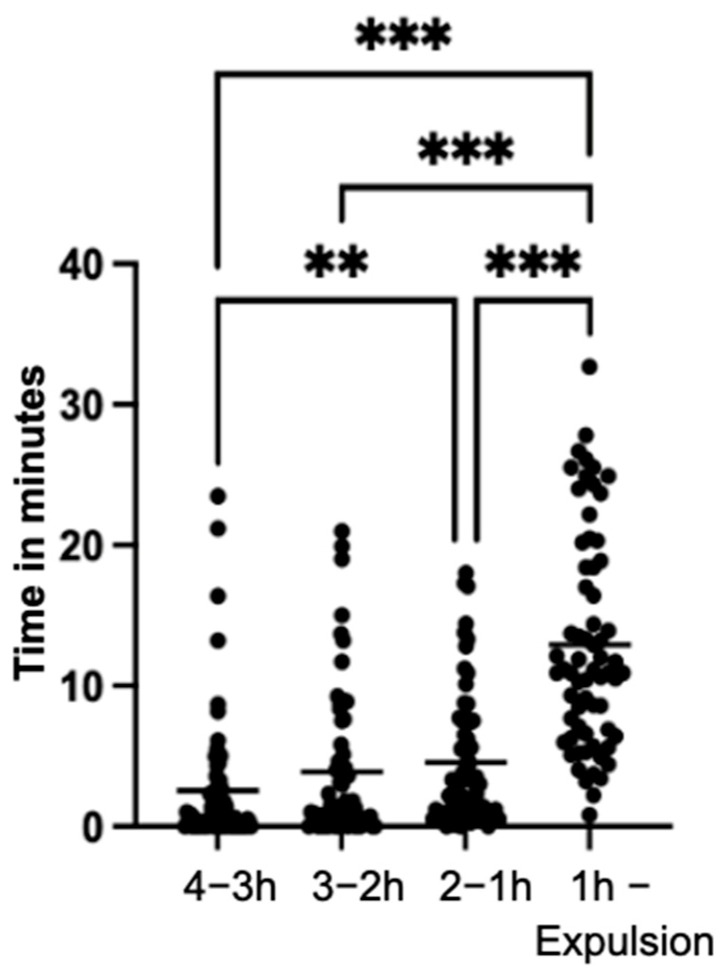
Duration and the mean value of the duration in minutes of the locomotor activity of the mares (n = 66), in the specified time intervals of the observation period (significance levels: **: *p* < 0.01; ***: *p* < 0.001).

The mares’ locomotor activity exhibited a highly significant increase (*p* < 0.001) during the last hour of the observation period compared to the previous intervals (4–3 h; 3–2 h; 2–1 h) (Figure 4).

When examining only the three time intervals of the last 30 min of the observation period individually, the duration of the mares’ locomotion was comparable, averaging 2.3 min per 10 min time interval. Notably, in the time interval four to three hours before parturition, 20 mares did not exhibit locomotion. However, during the last hour of the observation period, all mares displayed this behavior, representing a highly significant difference (*p* < 0.001) compared to the interval of four to three hours.

### 3.3. Feed/Water Intake Behavior

Food/water intake increased highly significantly (*p* < 0.001) from an average duration of 5.9 ± 7.9 min in the interval four to three hours to 11.5 ± 9.5 min in the final hour (1 h–expulsion) of the observation period. However, the food/water intake in the last 10 min decreased to 0.7 ± 1.5 min compared to the interval 30 to 20 min (*p* < 0.01) of 1.8 ± 2.5 min and to the interval 20 to 10 min (*p* < 0.05) of 1.5 ± 2.3 min. In this time interval, the number of mares that could be observed during feed/water intake becomes smaller. In the time interval 30 to 20 min, 62.1% of the mares were observed; in the last ten minutes of the observation period, the behavior was observed in 33.3% of the mares. This difference was significant (*p* < 0.01).

### 3.4. Resting Behavior

**Figure 5:**
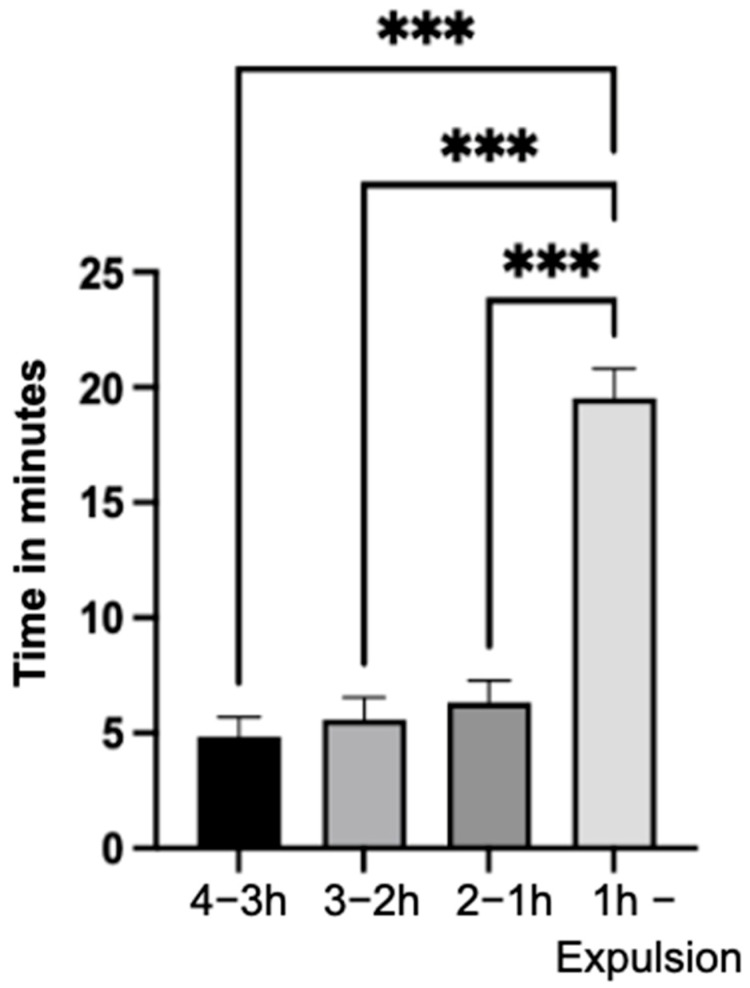
Resting behavior in minutes of the mares (n = 66) with indication of the standard error of the mean value in the specified time intervals of the observation period (significance level: ***: *p* < 0.001).

In the final hour of the observation period, the mares exhibited highly significantly (*p* < 0.001) longer resting behavior compared to preceding three time intervals (Figure 5). Within the last 30 min, the duration of resting behavior remained consistent across all mares (3.9 ± 2.5 min in the interval 30–20 min; 3.6 ± 2.8 min in the interval 20–10 min; 3.3 ± 2.5 min in the interval 10 min—expulsion). 

### 3.5. Defecation/Urination Behavior

The total number of repetitions of urination increased significantly (*p* < 0.001) from 0.6 ± 0.7 repetitions in the four to three hour interval to 1.3 ± 1.0 repetitions in the final hour of the observation period. Moreover, the number of mares observed urinating increased highly significantly (*p* < 0.001) from the interval from four to three hours compared to the interval from one hour to expulsion.

A highly significant (*p* < 0.001) increase from 0.6 ± 0.6 to 1.5 ± 1.3 repetitions was observed in the last hour of the observation period compared to the interval from four to three hours prior to parturition. Additionally, 50 mares defecated in the final hour of the observation period. This was more mares (*p* < 0.05) than in the four to three hours before parturition. 

### 3.6. Flehmen/Yawning/Empty Chewing

**Figure 6:**
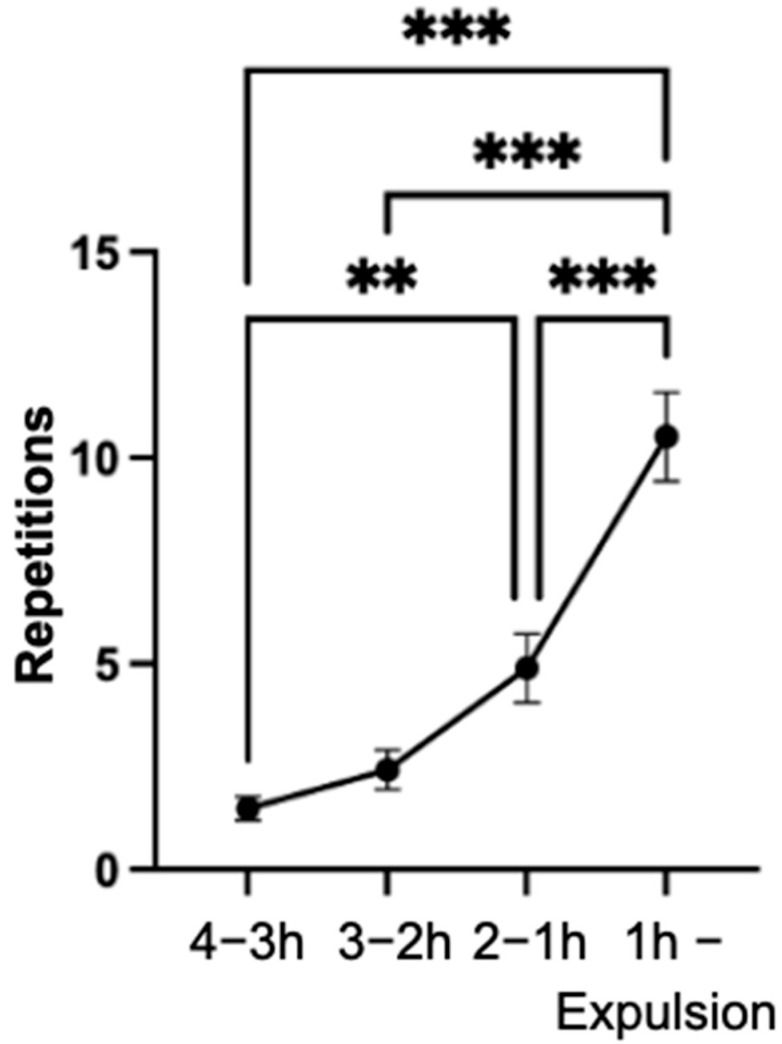
Mean values of the repetitions of the behavior flehmen/yawning/empty chewing of the mares (n = 66), with indication of the standard error of the mean value in the indicated time intervals of the observation period (significance levels: **: *p* < 0,01; ***: *p* < 0,001).

The total number of repetitions of flehmen, yawning or empty chewing significantly increased (*p* < 0.001) as the time of parturition approached (Figure 6). These behaviors were exhibited at least once and up to 47 times by 61 mares in the last hour before parturition. In 40 mares, the behaviors were observed in the last ten minutes of the observation period. 

### 3.7. Pawing with the Front Hooves

**Figure 7:**
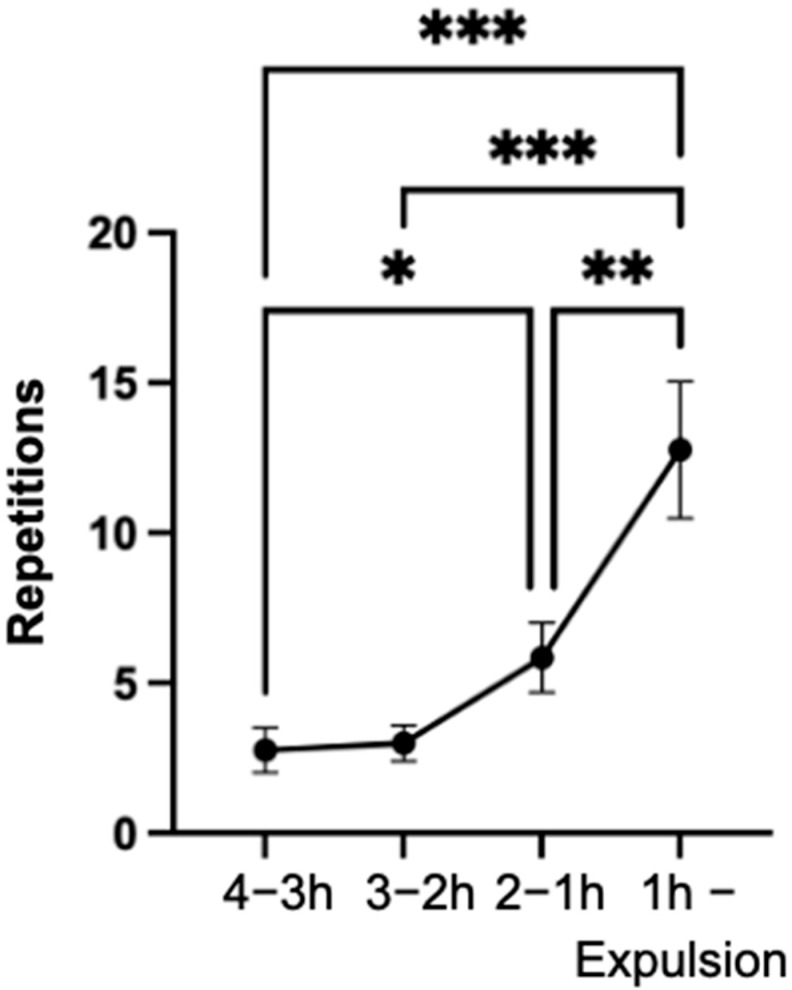
Mean values of the repetitions of the behavior of pawing with the mares’ front hooves (n = 66), with indication of the standard error of the mean value in the specified time intervals of the observation period. (significance levels: *: *p* < 0.05; **: *p* < 0.01; ***: *p* < 0.001).

The total number of repetitions of the pawing behavior with the front hooves increased significantly from the time intervals of four to three hours and three to two hours compared to the final hour of the observation period (*p* < 0.001) (Figure 7). However, a decrease in the mean values was observed from 2.8 ± 5.5 to 1.2 ± 1.9 repetitions when considering the time intervals of the last 30 min of the observation period separately. This difference was not statistically significant (*p* = 0.366).

In the final hour of the observation period, the behavior was observed in 86.4% of the mares; this increase was significantly different (*p* < 0.001) compared to the interval of four to three hours of the observation period.

### 3.8. Lifting the Tail

**Figure 8:**
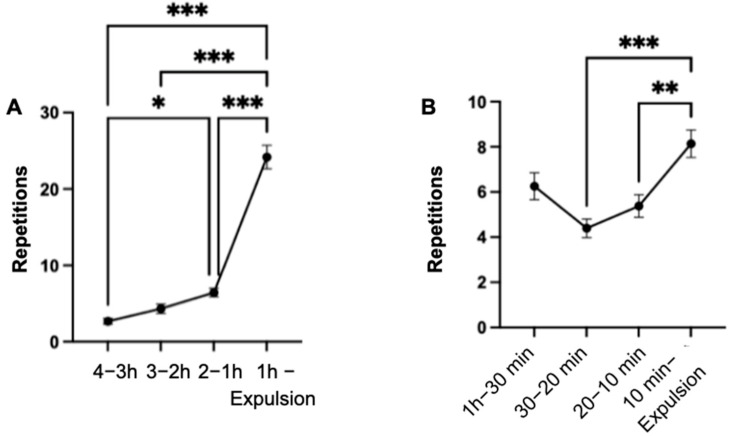
Mean values of the repetitions of the behavior lifting the tail of the mares (n = 66), with indication of the standard error of the mean value in the indicated time intervals (**A**) and in the last hour (**B**) of the observation period. (significance levels: *: *p* < 0.05; **: *p* < 0.01; ***: *p* < 0.001).

Lifting of the tail occurred significantly (*p* < 0.001) more frequently in the last hour compared to the three time intervals (4–3 h; 3–2 h; 2–1 h) of the observation period (Figure 8A). Additionally, it was observed that the tail was raised weakly significantly (*p* < 0.05) more frequently in hours two to one than in hours four to three of the observation period. 

In the final ten minutes of the observation period, the behavior was significantly (*p* < 0.001) more frequent among the mares compared to the interval of 30 to 20 min and significantly (*p* < 0.01) more frequent than in the interval of 20 to 10 min (Figure 8B). 

Throughout the last hour of the observation period, the behavior was observed at least four times and up to 66 times in all mares. The number of mares exhibiting the behavior significantly increased (*p* < 0.001) from the four to three hour interval to the last hour of the observation period.

### 3.9. Tail Slapping

The total number of repetitions of tail slapping behavior increased from the interval of four to three hours (7.3 ± 11.7 repetitions) compared to the last hour (10.5 ± 12.0 repetitions) of the observation period (*p* < 0.05). 

However, in the interval of 30 to 20 min, the total number of repetitions decreased from 1.9 ± 3.1 to 0.7 ± 1.3 repetitions in the last 10 min of the observation period (*p* < 0.01). 

The number of mares showing the behavior decreased from the interval of 30–20 min to the last ten minutes of the observation period (*p* < 0.05). 

### 3.10. Rolling 

**Figure 9:**
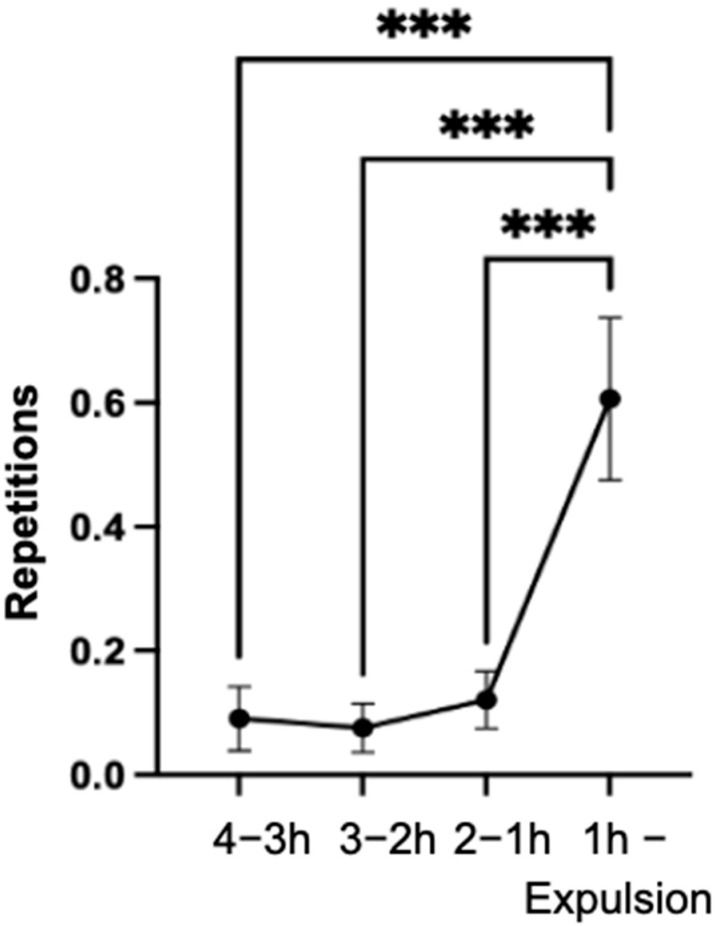
Mean values of the repetitions of the rolling behavior of the mares (n = 66) with indication of the standard error of the mean value in the specified time intervals of the observation period. (significance level: ***: *p* < 0.001).

The total number of repetitions of rolling increased significantly (*p* < 0.001) in the last hour of the observation period compared to the previous intervals (4–3 h; 3–2 h; 2–1 h) (Figure 9). The behavior was observed in 48.5% of the mares at least once during the entire observation period. Among the mares in which the behavior was observed, 62.5% exhibited the behavior only in the last hour of the observation period. Additionally, out of these 20 mares, the behavior was observed in 14 mares only during the last ten minutes. 

The number of mares exhibiting the behavior in the last hour was significantly higher (*p* < 0.001) compared to the number in the previous intervals (4–3 h; 3–2 h; 2–1 h) of the observation period.

### 3.11. Kicking with the Hind Legs towards the Abdomen

The mean value of repetitions increased highly significantly (*p* < 0.001) from the intervals of four to three hours (1.0 ± 1.9 repetitions) and three to two hours (1.6 ± 2.9 repetitions) compared to the last hour (4.6 ± 6.5 repetitions) of the observation period. Furthermore, a weakly significant (*p* < 0.05) increase was found when comparing the intervals of four to three hours and two to one hour before parturition.

When comparing the time intervals of 30 to 20 min and the last 10 min of the observation period, the total number of repetitions decreased weakly significantly (*p* < 0.05) from 1.4 ± 2.6 to 0.5 ± 1.2 repetitions.

Kicking with the hind leg to the abdomen was observed at least once in the last hour in 47 mares; this occurrence was significantly higher (*p* < 0.001) compared to the interval of four to three hours of the observation period.

### 3.12. Looking towards the Abdomen

**Figure 10:**
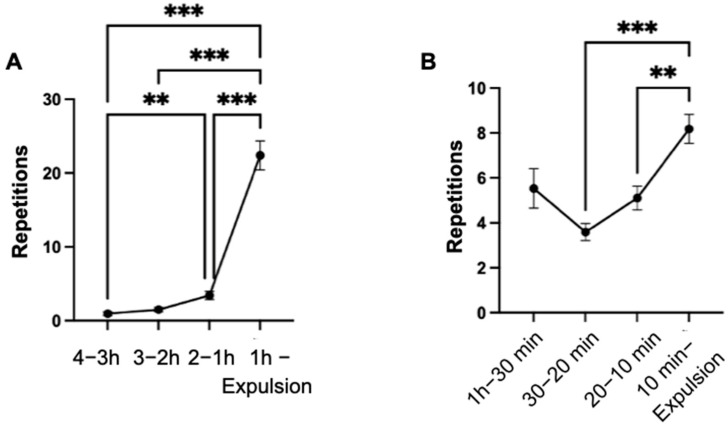
Mean values of the repetitions of the behavior of looking around at the abdomen of the mares (n = 66) with an indication of the standard error of the mean value in the indicated time intervals (**A**) and in the last hour (**B**) of the observation period (significance levels: **: *p* < 0.01; ***: *p* < 0.001).

There was a highly significant increase (*p* < 0.001) in the total number of repetitions of the behavior of looking towards the abdomen from the previous three intervals (4–3 h; 3–2 h; 2–1 h) compared to the last hour of the observation period (Figure 10A). Furthermore, a significant (*p* < 0.01) increase was found when comparing the intervals of four to three hours and two to one hour before parturition. There was a highly significant (*p* < 0.001) increase in the mean values for the 30 to 20 min interval compared to the last ten minutes of the observation period (Figure 10B). 

Ninety-seven percent of the mares exhibited the behavior in the last hour of the observation period, with all mares exhibiting the behavior less frequently in the previous three hours (4–3 h; 3–2 h; 2–1 h) of the observation period than in the last hour. The number of mares displaying the behavior in this period was highly significantly higher than in the previous three time (4–3 h; 3–2 h; 2–1 h) intervals (*p* < 0.001).

In 94% of the mares, the behavior was observed at least once in the last ten minutes of the observation period. 

### 3.13. Rubbing the Rear Body Region/Perineal Area against the Stall Wall

The total number of repetitions of the rubbing of the rear body region increased only slightly from 0.5 ± 1.3 repetitions in hours four to three to 1.7 ± 3.9 repetitions in the last hour of the observation period (*p* = 0.71). Twenty-seven mares showed this behavior in the last hour of the observation period, although there was no statistically significant difference compared to the previous time intervals (4–3 h; 3–2 h; 2–1 h) (*p* = 0.111).

### 3.14. Influence of Parity and the Birth Process

The total number of repetitions of lying in the sternal position was significantly different (*p* < 0.05) between the maiden mares with 1.0 ± 1.5 repetitions and the pluriparous mares with 2.1 ± 1.8 repetitions in the last hour of the observation period. 

A significant difference could not be detected for any other behavioral domain. 

Mares that developed dystocia exhibited lateral recumbency in the last hour of the observation period with 2.5 ± 3.2 repetitions, weakly significantly (*p* < 0.05) more frequently than mares with eutocia with 1.0 ± 2.5 repetitions. There were no other differences between the groups.

## 4. Discussion

Ensuring that the parturition proceeds without a problem and monitoring the timely intake of colostrum by the foal [4,5,17] is a basic prerequisite for the development of a healthy foal. The relatively short second stage of parturition, the expulsion phase, lasting 20–30 min [18], severely limits the time window in which successful birth assistance can be provided. Therefore, problems must be recognized quickly. Dystocia is present in two to thirteen percent of mare parturitions [7] and is considered an absolute emergency in horses, in which the immediate recognition and taking of appropriate obstetric measures is of crucial importance. 

The high variability of gestation length in mares [1] and the poor usability of externally visible physical changes to predict the time of parturition, which vary significantly among individuals in expression and timing [2,3], and the fact that most foals are born during the night [19], complicate birth monitoring. Therefore, the use of birth monitoring systems is of central importance.

Various birth detection systems have been developed to relieve the burden on humans during birth monitoring [6,10,12,14,20]. A detailed description of maternal behavior in the last hours before foal expulsion can help improve existing birth monitoring systems or to develop new ones. 

In the present study, the behavior of the mare in stage I of parturition was specifically addressed and examined in detail in order to be able to depict the specific behavior of the mares during parturition. The aim of further studies is to compare the behavior observed in this study with the behavior of mares in the phase of late gestation and in the postpartum period in order to be able to establish even more precisely specific behaviors that are exclusively associated with stage I of parturition.

In this study, mares of the same breed kept under the same housing conditions were included. Differences in the physiology of mares in the postpartum period have been described in the literature for different breeds [17], but no breed-specific differences in behavior have been described at this time to our knowledge. This aspect requires further research.

Although the study was carried out in a veterinary practice, an influence on the behavior of the mares can be ruled out by clearly separating the stables for the broodmares, where the parturition also takes place.

Several authors [2,9,10,15] have investigated the behavior of mares during parturition, but no study has continuously observed a comparably large group of animals in the hours preceding foal expulsion. Since most mares give birth lying down and rarely exhibit lying behavior during late gestation [15], lying down is a behavior that changes in most mares as parturition approaches. In the present study, a highly significant increase in the duration of lying in the sternal position was observed in the last ten minutes before parturition. The duration of lying in the lateral position also increased significantly. A significant increase in lying time was also observed by Bachmann et al. [14] immediately before parturition. Auclair-Ronzaud et al. [10] also observe an increase in the duration of lying in the lateral position closer to the time of parturition. Other authors also observe an increase in lying behavior in this period [9,11,15], but do not make any statement about the duration of the behavior observed. Additionally, they do not specify the time interval in which these changes occurred. All authors who observe the lying behavior of mares before parturition describe strong individual differences between the mares [9,14], which were also evident in the present study. Considering the individual differences observed and the instances where some mares in the present study exhibited no lying behavior throughout the entire observation period, with the foal’s expulsion occurring in a standing position, it is evident that lying behavior may not serve as a reliable indicator of parturition in all mares. When evaluating lying behavior, none of the authors mentioned above make a distinction between the influences of parity and the birth process. In this study, it was shown that the mares that developed dystocia were more likely to lie laterally in the hour before parturition than mares with eutocia. It must be emphasized that no other behavioral differences were found between mares with eutocia and dystocia in stage I of parturition. In a comparable study in cattle, the behaviors of wall rubbing, frequency of urination, and floor pawing differ between cattle with eutocia and dystocia in stage I of parturition [21]. It should also be noted that maiden mares showed the behavior of lying in the sternal position less frequently compared to pluriparous mares. Lying laterally was only shown by one maiden mare in the last hour before parturition. For the evaluation of behavioral parameters in stage I of parturition, parity influences should therefore not be excluded and should be analyzed in further studies.

It has been shown that the duration of the mares’ locomotor activity increases significantly in proximity to the time of parturition. This has already been described by other authors [2,12,13,14,15]. Although the change in locomotor activity closer to the time of parturition has been described by many authors and could be observed in the present study, there is, to our knowledge, currently no commercially available birth monitoring system based on the change in this behavior. The pilot studies by Bachmann et al. [14], Hartmann et al. [12], and Borchers [11], which measure the increase in locomotor activity with the help of sensors, show the potential that lies, in principle, in the modification of this behavior for birth detection. However, all authors describe clear individual differences in the locomotor activity of the mares, which could also be observed in this study. Developing an individual locomotion profile and measuring changes in proximity to the time of parturition appears to be a sensible approach for use as a birth monitoring system [14].

Jeffcott [2] observed that the mares stop eating in proximity to the time of parturition. However, in the present study, an increase in the average duration of feed and water intake was observed over the entire observation period approaching parturition. Shaw et al. [9] observe that the feed intake behavior of the mares on the night of parturition did not change compared to the average values of the previous days and that a significant decrease in behavior was only observed in the 30 min before parturition. This observation is consistent with the results of the present study, which also found a decrease in the time spent on feed/water intake in the 30 min before parturition. Jung et al. [15] also observed a significant reduction in the mares’ feed intake behavior in this time window compared to the previous days. This time window roughly coincides with the duration of stage I of parturition. The onset of stage I of parturition and the associated abdominal pain [2,9] can explain the cessation of feed intake in proximity to the time of parturition. 

Jung et al. [15] demonstrate that a larger number of mares exhibit resting behavior in the last hour before parturition than in the previous days, with a significant increase observed in the 30 min before parturition. This finding aligns with observations in the present study, where a highly significant increase in the duration of resting behavior was documented in the last hour of the observation period compared to previous intervals. However, no increase was observed when the last 30 min of the observation period were considered separately. Jung et al. [15] attribute the increased resting behavior to decreasing feed intake, a factor not fully supported by the present study’s findings in the last hour of observation.

Conversely, Shaw et al. [9] observed a significant decrease in resting behavior closer to the time of parturition. They attribute this observation to increased locomotor activity and lying behavior during this period. The disparity with the present study’s results stems from the methodology differences: while the present study recorded and evaluated every behavior shown within the observation interval, Shaw et al. [9] recorded only one behavior at fixed points in time within the observation interval. 

The observation in the present study of the accumulation of resting behavior in the approach to the time of parturition contrasts with the observed increased locomotor activity of the mares in the same time phase. However, the evaluation method employed does not clearly indicate in the results that the mares frequently switch between individual behaviors such as walking, eating, and resting as they approach parturition, as only the total duration of the behavior shown is provided in the study. This gives the impression that the mares were resting for a long time in the last hour of the observation period. However, this was not the case, as the mares frequently switched between the behaviors, which describes the mares’ restless basic behavior in proximity to the time of parturition. 

Defecation and urination are part of the normal behavioral repertoire of a horse, and alterations in these behaviors are not typically considered specific to a mare before parturition [15]. Nevertheless, both Jung et al. [15] and Jeffcott [2], as well as the present study, observed an increase in the total number of repetitions of defecation in proximity to the time of parturition. Even though a highly significant increase in total number of repetitions was observed in the last hour of the observation period in the present study compared to the previous intervals, it was not reliable for predicting the time of parturition. This is partly due to the low average number of defecation repetitions. The same trend applies to urine output, with a highly significant increase in total number of repetitions but also characterized by a low number of repetitions, rendering it unsuitable for predicting parturition. Jung et al. [15], who also observed increased urination behavior in the hour before parturition, reached the same conclusion.

A highly significant increase (*p* < 0.001) in repetitions over the observation period was observed for the flehmen, yawning and empty chewing behavior. Auclair-Ronzaud et al. [10] also observed this behavior; however, they were also able to determine an increase in the duration and total number of repetitions as the time of parturition approached. Frequent flehmen, yawning, or even empty chewing are behaviors that can be associated with the occurrence of abdominal pain [22,23,24]. The data collected reflect this observation. The highly significant increase in repetitions and that the behavior was exhibited by almost all mares in the last hour of the observation period underlines the statement. Surprisingly, the total number of repetitions was not influenced by the presence of dystocia. The three behaviors described were summarized in the present study based on the work of van Loon and van Dierendonck [23], who also summarize flehmen and yawning in their study on the pain behavior of horses. In further studies, it would be interesting to differentiate the behaviors in order to be able to assess their individual influence.

A highly significant increase (*p* < 0.001) in the average repetitions of the pawing behavior was observed closer to the time of parturition, with a subsequent decrease in the last 30 min. Jung et al. [15] observed that more mares exhibited the behavior of pawing with the front hooves in the last hour before parturition compared to the previous days. In the present study, the behavior was also observed at least once in 86.4% of the mares in the last hour of the observation period. This suggests that pawing with the front hooves exhibited more frequently by mares due to the onset of abdominal pain during parturition. This aligns with the observations by Sutton et al. [22] and van Loon and van Dierendonck [23], who describe frequent pawing with the front hooves as a behavior associated with the onset of abdominal pain. Whether the behavior can be measured using sensors warrants further investigation.

In this study, a highly significant increase in the total number of repetitions of tail lifting was observed in proximity to the time of parturition. The behavior was observed in all mares in the last hour of the observation period, and all mares also showed this behavior in the last ten minutes. Auclair-Ronzaud et al. [10] similarly note changes in tail posture and movement as parturition approached, suggesting a positive correlation between increased tail movement and the onset of parturition. However, they do not differentiate between tail lifting and tail slapping. Using an accelerometer, they measure the duration and frequency of tail movements and proposed its potential use in mare birth monitoring. They observe that the duration of tail movements decreased while the frequency increased in proximity to the time of parturition. The results of the present study regarding tail lifting suggest its potential suitability for predicting the time of parturition in mares. Sensor-based birth detection, including one attached to the animal’s tail, has been explored in cow birth monitoring [25,26]. Additionally, a commercially available birth detector for cows has been tested by Górriz-Martín et al. [25].

Less than half of the mares exhibited rolling, indicating that rolling is not specific to the onset of parturition in mares. 

Regarding the behavior of kicking with the hind legs to the abdomen, a highly significant (*p* < 0.001) increase in the average repetitions was observed in proximity to the time of parturition. Forty-seven mares exhibited this behavior in the last hour of the observation period. While not described as indicative of parturition, kicking with hind legs toward the abdomen is considered by various authors to signal abdominal pain [22,23,24]. Sutton et al. [22] substantiate the description that the more frequently and violently horses kick with their hind legs towards the abdomen, the stronger the underlying abdominal pain. The significant increase in behavior in proximity to the time of parturition can be explained by the increase in abdominal pain due to the onset of parturition and the associated myometrial and abdominal contractions. 

The majority of mares exhibited the behavior of looking towards the abdomen in the last hour of the observation period. Notably, none of the mares exhibited this behavior more frequently in previous time intervals than in the last hour. Borchers [11] also notes this behavior before parturition, while Jung et al. [15] describe a non-significant increase in the number of mares in the last hour before parturition compared to the previous comparison days. Auclair-Ronzaud et al. [10] describe looking towards the abdomen as part of a specific behavioral complex exhibited by mares in the prepartum phase, noting its increased frequency as parturition approached. Sanchez and Robertson [27] and Hernández-Avalos et al. [24] rate looking around at the abdomen as a clear sign of abdominal pain, while Sutton et al. [22] rate it as a sign of mild abdominal pain. In the present study, the highly significant increase in the mean values of the repetitions clearly showed that this is a behavior that can be associated with parturition. The behavior can be explained by the abdominal pain caused by the onset of parturition. The different number of repetitions shown by the mares in the last hour of the observation period may indicate that the mares react differently to the pain caused in the context of parturition.

Bachmann et al. [14] show that the movements of the head/neck, measured with the aid of sensors, increase significantly in the prepartum period. 

Further studies should investigate whether the behavior can be measured using sensors attached to the mare’s halter and whether the behavior is specific enough to predict parturition. 

Rubbing of the rear body region against the stall wall was observed in 41% of the mares in the last hour of the observation period. The number of mares exhibiting this behavior and the total number of repetitions of this behavior increased in proximity to the time of parturition. Borchers [11] also observe this behavior in mares. Auclair-Ronzaud et al. [10] describe the pressing or rubbing of the hindquarters against the stall wall as a typical behavior of mares before parturition and are able to determine an increase in the behavior in proximity to the time of parturition. Since 59% of the mares did not show the behavior in the last hour of the observation period, it is not suitable for predicting the time of parturition. 

## 5. Conclusions

In this study, the behavioral patterns of mares in the last four hours before parturition were examined. Due to the strong individual characteristics of the behaviors, a combination of different behaviors should be recorded for automated birth monitoring. Looking towards the abdomen, lifting the tail, as well as flehmen, yawning, or empty chewing, along with changes in locomotor activity, appear to be suitable indicators.

Lying behavior is already integrated into birth detectors available on the market for birth monitoring. The findings of this study further support the use of lying behavior in detecting parturition in mares. However, it is important to note that not every parturition would have been detected solely based on lying behavior in this study. Additionally, the onset of this behavior, which sometimes occurs shortly before expulsion of the foal, limits its utility for birth monitoring.

## Data Availability

The data presented in this study are available upon request of the corresponding author.
